# Three-Dimensional Printing Multi-Drug Delivery Core/Shell Fiber Systems with Designed Release Capability

**DOI:** 10.3390/pharmaceutics15092336

**Published:** 2023-09-18

**Authors:** Hao Wei, Yongxiang Luo, Ruisen Ma, Yuxiao Li

**Affiliations:** 1 Marshall Laboratory of Biomedical Engineering, Shenzhen University, Shenzhen 518060, China; weihao5039@163.com (H.W.); luoyongxiang@szu.edu.cn (Y.L.); 2 Guangdong Key Laboratory for Biomedical Measurements and Ultrasound Imaging, Department of Biomedical Engineering, Shenzhen University Medical School, Shenzhen 518055, China; ruisenma@163.com; 3 College of Biological and Chemical Engineering, Qilu Institute of Technology, Jinan 250200, China

**Keywords:** 3D printing, multi-drug delivery, core/shell fibers, on-demand release

## Abstract

A hydrogel system with the ability to control the delivery of multiple drugs has gained increasing interest for localized disease treatment and tissue engineering applications. In this study, a triple-drug-loaded model based on a core/shell fiber system (CFS) was fabricated through the co-axial 3D printing of hydrogel inks. A CFS with drug 1 loaded in the core, drug 2 in the shell part, and drug 3 in the hollow channel of the CFS was printed on a rotating collector using a co-axial nozzle. Doxorubicin (DOX), as the model drug, was selected to load in the core, with the shell and channel part of the CFS represented as drugs 1, 2, and 3, respectively. Drug 2 achieved the fastest release, while drug 3 showed the slowest release, which indicated that the three types of drugs printed on the CFS spatially can achieve sequential triple-drug release. Moreover, the release rate and sustained duration of each drug could be controlled by the unique core/shell helical structure, the concentration of alginate gels, the cross-linking density, the size and number of the open orifices in the fibers, and the CFS. Additionally, a near-infrared (NIR) laser or pH-responsive drug release could also be realized by introducing photo-thermal materials or a pH-sensitive polymer into this system. Finally, the drug-loaded system showed effective localized cancer therapy in vitro and in vivo. Therefore, this prepared CFS showed the potential application for disease treatment and tissue engineering by sequential- or stimulus-responsively releasing multi-drugs.

## 1. Introduction

Many severe diseases generally require synergistic treatment with multiple drugs because of their complex pathological conditions. The multi-drug system has been widely used for cancer treatment [1,2,3] and has become a promising approach for the treatment of complex disease conditions, such as the prevention of bacterial infections [4] and anti-inflammation [5]. Conventional administration methods, such as oral administration and intravenous injection, generally face significant challenges, including strong toxic side effects, low drug therapeutic efficacy, a deficient delivery mechanism at the target site, poor bioavailability and nonspecific biodistribution, drug overdose, and drug resistance [6,7,8]. Local multi-drug delivery is a promising strategy for overcoming these challenges because of the direct-targeted characteristics that improve efficacy and minimize the distribution and side effects of drugs in the whole body. Common drug nano/micro carriers, such as nanocapsules [9], nanoparticles [10,11], nanomicelles [12], etc., can achieve topical administration while also being rapidly ingested and massively cleared by the mononuclear phagocyte system in the body. Therefore, these nano/microcarriers are not suitable for local implantation applications.

In contrast, drug-loaded scaffolds and implants based on “smart” materials, such as core/shell fibers and scaffolds, are highly stable physiochemically and biochemically [13,14]. Drugs were loaded in the core part, enclosing it with a layer of shell materials to decrease the free diffusion of the drugs from the system, achieving sustained release. Drug release through this delivery system can also be controlled directly by an interaction between the “smart” material and changes in its environment, which build the remote-triggering drug release mode and provide the possibility of flexible control in the on-demand site, dose magnitude, and timing. Among all materials, hydrogels are the promising candidates for multi-drug delivery due to their high drug-loading efficiency, strong plasticity, and excellent biocompatibility [15,16,17]. Drugs can be released from hydrogels in response to different environmental stimuli, such as light [18], temperature [19], pH [20], salt concentration [21], magnetism [22], etc. Specifically, environmental stimuli-driven drug delivery holds the advantages of noninvasive, spatiotemporal, and remote-control characteristics, allowing for on-demand release for the treatment of specific diseases, such as protecting the drugs from degradation in the gastrointestinal tract and delivering drugs to the target lesion location. Light and pH had both received considerable attention for controlled drug delivery because they possessed spatial and temporal control over the release, high biocompatibility, and convenience. An optical stimulus could trigger a photoinduced transition of hydrophobicity to hydrophilicity, photolysis, photo rearrangement reaction, or photo-thermal reactions, allowing the time and location of drug release to be determined [23]. Furthermore, the change in pH in the surrounding environment could induce the gel-to-sol-phenomenon, which causes drug release on demand. The pH-responsive hydrogels were of great attraction for developing pH-responsive tumor-specific targeted drug delivery systems [24]. In our previous study, 3D-printed near-infrared (NIR) light-responsive core/shell fibers for in situ injection were developed using polydopamine (PDA)/alginate (Alg) as the shell parts and drug-loaded temperature-sensitive gelatin hydrogels as the core parts [25]. The drugs loaded in the core parts of the fibers achieved controlled release in an on-demand manner through the thermo-responsive reversible sol-to-gel transition of the hydrogel. However, most of these reported hydrogel systems are generally designed for the delivery of one type of drug. The hydrogel system delivery of multiple drugs with a designed release manner still needs further investigation.

Herein, we prepared a core/shell fiber system (CFS) with triple-drug loading in different locations of the CFS via co-axis 3D spiral printing to achieve the sequential release of multiple drugs. Furthermore, the multi-drug delivery capacity and the effect of multi-drug combination therapy on the CFS were evaluated while using common cancer treatment drugs (for example, DOX and PXT). The results showed that the CFSs loading DOX and PXT had a better ability to kill cancer cells. The synergistic effect of DOX and PXT exhibited excellent antitumor efficiency toward various solid tumors, such as breast cancer, by blocking off cells in the G2 m phase [26,27]. Furthermore, polydopamine or methacrylic acid copolymer-modified polycaprolactone (MAC−PCL) could also be introduced into this system to achieve NIR-triggered or pH-responsive drug release. The CFS is expected to achieve the goal of “personalized medicine,” in which each patient can customize their own private combination of drug therapies to realize maximum efficacy while reducing dosage awareness and the possible development of drug resistance.

## 2. Materials and Methods

### 2.1. Materials

Sodium alginate and gelatin were purchased from Sigma–Aldrich (St. Louis, MO, USA). Dopamine hydrochloride (DA, 98%) and Tris(hydroxymethyl) aminomethane (extra pure, 99.5%) were obtained from J&K scientific Ltd. (Shanghai, China). Methacrylic acid copolymer type C (Eudragit L100-55, MAC) was from yingdemaofeng pharmaceuticals Co. Ltd. (Guangdong, China). Polycaprolactone (PCL), 1-Hydroxybenzotriazole (HOBT), and dimethylformamide (DMF) were purchased from Macklin Chemical Reagent Co. Ltd. (Shanghai, China). Doxorubicin hydrochloride (DOX·HCl), paclitaxel (PTX), and 1-Ethyl-3-(3-dimethlaminopropyl)-carbodiimide hydrochloride (EDCI) were obtained from Aladdin Chemical Reagent Co. Ltd. (Shanghai, China). Dulbecco’s modified Eagle medium (DMEM), penicillin, Trypsin-EDTA solution, and sterilized fetal bovine serum (FBS) were purchased from Solarbio (Beijing, China). Propidium iodide solution was purchased from BioLegend (San Diego, CA, USA). Mouse breast cancer (4T1) cells were obtained from Cyagen (Shanghai, China). Cell Counting kit-8 was obtained from ZomanBio (Beijing, China).

### 2.2. Preparation and Characterization of Drug-Loaded Core/Shell Fiber Capsules

First of all, alginate solutions with different concentrations were prepared as the printing inks with selective addition of DOX (solution: deionized water, 1%, *w*/*v*). Then, the inks were loaded into three independent printing tubes equipped with printing nozzles. Ink with alginate concentrations of 5 wt%, 7 wt%, or 9 wt% were printed as the core and 9 wt%, 12 wt%, or 15 wt% as the shell part of the core/shell fibers using co-axial 3D printing with GUI-based control software (BioScaffolder 3.1, Gesim, Germany). The core/shell fibers were printed on the collector of a motor-driven roller. The printing speed was 6 mm/s, and the pressure was around 200–300 kPa. Afterward, the third ink with an alginate concentration of 5 wt%, 7 wt%, or 9 wt% was printed into the chamber of the core/shell fiber capsules. Finally, the prepared core/shell fiber capsules were cross-linked by 1 M CaCl_2_ solution, followed by washing with deionized water three times for 5 min each.

PLGA microspheres were prepared by the water-in-oil-in-water technique. Briefly, 100 mg PLGA and 10 mg dexamethasone (DEX) were dissolved in 500 μL chloroform. The mixture solution was mixed with 2 mL PVA solution (2%, *w*/*v*) and homogenized for 2 min. This emulsion was then added to 30 mL of PVA solution (0.2%, *w*/*v*) and stirred for 4 h. The microspheres were then collected via centrifugation (11,000 rpm at 4 °C) for 10 min. The PLGA microspheres were then washed twice with deionized water and centrifuged under the previously stated conditions. The prepared microspheres were freeze-dried for 48 h and mixed into the alginate inks to print as the shell of the core/shell fiber capsules.

The microstructures of the core/shell fiber capsules and PLGA microspheres were observed by field emission scanning electron microscopy (SEM, ZEISS SUPRA^®^ 55, Carl Zeiss, Oberkochen, Germany). After drying at room temperature, all samples were sputtered with gold. The specimen was then imaged in SEM operated at 5 kV with probe currents of 0.1 nA.

### 2.3. In Vitro Drug Release of the Core/Shell Fiber Capsules

To study the in vitro release of DOX from the core/shell fiber capsules, the DOX-loaded (1%, *w*/*v*) core/shell fiber capsules were incubated in 2 mL phosphate-buffered saline (PBS, pH 7.4). At each time point, 1 mL PBS was taken to measure the concentration of DOX by UV–Vis absorption spectra (Cary 60 UV, Agilent Technologies, Santa Clara, CA, USA) at 485 nm. Then, 1 mL of fresh PBS was added to each sample. 

DEX release profiles were determined by suspending drug-loaded particles (0.08 g) and core/shell fiber capsules carrying the drug-loaded microspheres (0.08 g) in 50 mL of PBS, respectively. The samples were incubated at 37 °C under agitation (50 rpm). At selected time points, the supernatants were removed and replaced with fresh buffer. The concentration of DEX in the supernatant was determined using the UV detection method described above. 

### 2.4. The Core/Shell Fiber Capsules with NIR-Triggered on-Demand Drug Release 

Firstly, the gelatin printing inks were prepared according to the following steps. The gelatin (5 g) and the DOX (100 mg) were added to 10 mL of deionized water at 60 °C, and the mixture was stirred vigorously until the powder was completely dissolved. DA solution was prepared by dissolving 100 mg DA into 20 mL Tris-HCl solution (100 mM, pH 8.5). Then, 3.6 g alginate was added to 20 mL DA solution, and the mixture was stirred vigorously until the homogeneous paste was produced. The prepared Alg-PDA inks and gelatin inks were printed as the shell and core part of the core/shell fibers capsules by injection and extrusion-based 3D printing with GUI-based control software (BioScaffolder 3.1, Gesim, Germany). Co-axial core/shell nozzles with different sizes can be used to make core/shell fiber capsules with different diameters. Afterward, the prepared core/shell fiber capsules were cross-linked by soaking in 1 M CaCl_2_ solution for 5 min, followed by washing with deionized water three times.

To study the in vitro release of DOX from core/shell fiber capsules, the PDA/Alg and DOX-loaded (1%, *w*/*v*) hydrogel core/shell fiber capsules were incubated in 2 mL PBS and then irradiated by 808 nm NIR laser for 3.5 min, followed by a 10 min interval. Five cycles were performed. The drug-loaded core/shell fiber capsules without laser irradiation were performed as a control. After each period of irradiation, 1 mL of solution was taken to measure the concentration of DOX, and another 1 mL of fresh solution was added to each sample. The concentration of DOX was determined by UV–Vis absorption spectra (Cary 60 UV, Agilent Technologies, Santa Clara, CA, USA) at 485 nm. The temperature of core/shell fiber capsules under NIR irradiation was determined by an infrared thermal camera (FLIR A310, Sweden).

### 2.5. In Vitro Cancer-Cell Treatment by Using Dual-Drug-Loaded Core/Shell Fiber Capsules

Cells (4T1) were seeded into 24-well plates at a density of 1 × 104 cells per well and cultured in DMEM (10% fetus bovine serum, 1% penicillin, 1% glutamine, and 1% sodium pyruvate) at 37 °C with 5% CO2 for 24 h. Afterward, the core/shell fiber capsules were added to the wells and treated in four different groups: (1) DOX (1%, *w*/*v*) loaded in a hollow pipe of the alginate core/shell fiber capsules; (2) the alginate core/shell fiber capsules were loaded with PTX (1%, *w/v*); (3) DOX (1%, *w*/*v*) and PTX (1%, *w*/*v*) carried out simultaneously; (4) no treatment (control). After incubation for another 12 h, the cell viability was measured by CCK-8 cell cytotoxicity assay. Calcein-AM and propidium iodide were used for live/dead cell staining, and the images were observed by a Nikon Eclipse Ti inverted fluorescence microscope (Nikon Canada, Mississauga, Canada). Annexin V-FITC/PI Apoptosis Detection Kit was used to detect cell apoptosis, and the results were determined by flow cytometry (CytoFLEX, Beckman Coulter, Brea, CA, USA).

### 2.6. In Vivo Cancer Therapy by Using Dual-Drug-Loaded Core/Shell Fiber Capsules

The animal experiment was approved by the Animal Welfare and Research Ethics Committee at Shenzhen University (A202201445). To obtain subcutaneous tumors, 4T1 cells (1 × 106 cells in 100 mL DMEM) were injected into each mouse (Balb/C female, 3–4 weeks old). When the tumor volume reached a size of about 200 mm^3^, all mice were randomly divided into four groups and treated as previously described in Section 2.5. The core/shell fiber capsules with the size of 5 mm in length and 1 mm in diameter were implanted into the tumor. All mice were anesthetized by intraperitoneal injection of 10% chloral hydrate before surgery. For 15 days after the corresponding treatments, the volume of tumors and body weight were measured every two days. In addition, the major organs (heart, liver, spleen, lung, and kidney) were sectioned into slices and stained with hematoxylin and eosin (H&E) for histological analysis.

### 2.7. The Core/Shell Fiber Capsules with pH-Responsive Drug Delivery 

The MAC−PCL was prepared by an esterification reaction of the hydroxyl group in PCL and the carboxylic acid group in MAC. The reaction between MAC and PCL was conducted in the presence of EDCI and HOBT in DMSO. In a dried flask, MAC (2 g), EDCI (0.16 g), and HOBT (0.12 g) were dissolved in 20 mL anhydrous DMF and stirred for 30 min at room temperature. Then, PCL (0.8 g) was added, and the mixture solution was stirred for 48 h at room temperature. The product was dialyzed against water for 48 h. After lyophilization for 24 h, MAC−PCL polymer was obtained. Using D-DMSO as solvents, 1H NMR spectra of MAC−PCL polymer were recorded on a nuclear magnetic resonance instrument (AVANCE Ⅲ 500MHZ). FTIR spectra of a MAC-g-PCL polymer were obtained from a spectrometer (spectrometer) using the KBr pellets method by scanning from 4000 to 500 cm^−1^. 

The water absorption of hydrogels was studied in solutions with different pH values (pH 1.2, pH 4.6, and pH 7.4). At proper intervals, hydrogels were taken out from the solutions, and the water absorption was calculated. After swelling to equilibrium in different solutions (pH 1.2 and pH 7.4), dried hydrogels were obtained by lyophilization for 24 h. The morphology was observed using electron microscopy (SEM, ZEISS SUPRA^®^ 55, Carl Zeiss, Oberkochen, German). 

The vancomycin (100 mg) was dissolved in 10 mL deionized water, and then MAC−PCL hydrogel (2 g) was added into the solution and kept there for 4 h. Then, the vancomycin-loaded MAC−PCL inks were printed in the hollow channel of the core/shell fiber capsules. The release of vancomycin from core/shell fiber capsules was studied using the dialysis method at different pHs (pH 1.2 and pH 7.4). The dialysis membrane with MWCO 1000 containing 10 mg of drug-loaded hydrogel in core/shell fiber capsules was immersed in a 30 mL medium at 37 °C. At appropriate time intervals, 10 mL of PBS medium was removed and replaced with equivalent PBS. The concentration of vancomycin was determined by UV–Vis absorption spectra at 220 nm.

### 2.8. Statistical Analysis

All results in this study were obtained from at least three duplicate samples and exhibited as mean ± standard deviation using one-way ANOVA analysis.

## 3. Results and Discussion

### 3.1. Preparation of Triple Drugs Loaded System and the Release of the Drugs In Vitro 

**Figure 1:**
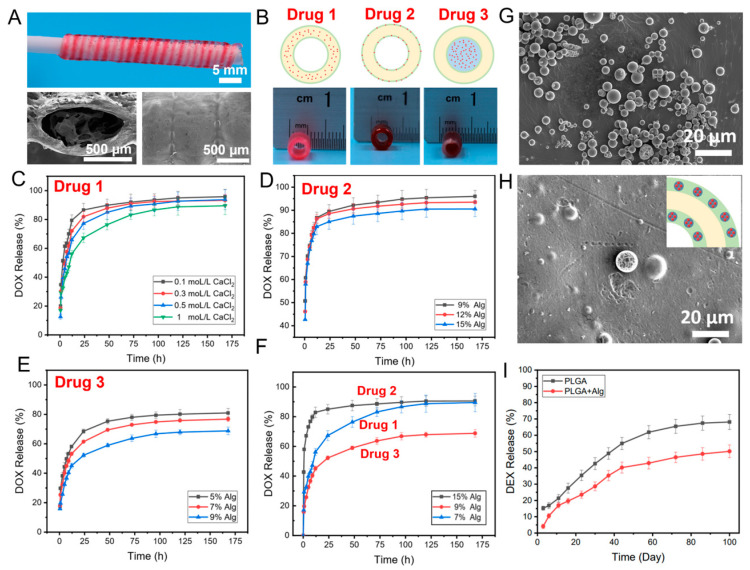
The photograph and SEM images of the fabricated CFS (**A**); the schematic diagram and photographs of drugs loading in the core (drug 1), shell (drug 2), and channel (drug 3) of the CFS (**B**); DOX release from the core part (drug 1) of the CFS cross-linked with different concentrations of CaCl_2_, *n* = 4 (**C**); DOX release from the shell layer (drug 2) of the CFS with different concentrations of alginate, *n* = 4 (**D**); DOX release from the channel (drug 3) of the CFS with different concentrations of alginate, *n* = 4 (**E**); DOX release from different part of the CFS, *n* = 4 (**F**); SEM images of the prepared PLGA microspheres (**G**) and PLGA microspheres embed in alginate shell of CFS (**H**); and DEX release from microspheres in the shell layer of the CFS, *n* = 4 (**I**).

A multiple-drug co-delivery system with controlled release capability is important for tissue engineering and disease treatment [28,29]. Herein, we fabricated a co-delivery system with triple drugs loaded spatially using 3D printing. A core/shell fiber system (CFS) with drug 1 loaded in the core and drug 2 in the shell part was printed on a rotating collector using a co-axial nozzle (Figure 1A). The concentration of the alginate is the key factor for the fabrication of the core/shell fibers [25]. In our previous study, we demonstrated that the alginate inks with concentrations around 4–7 wt% and 9–18 wt% were suitable for 3D printing of the core and shell part of the core/shell fiber scaffolds, respectively [25]. From the SEM image, the surface of the fiber was dense without pores, which could reduce the drug leakage from the fiber. After printing, the CFS was removed from the collector, and alginate inks containing drug 3 were printed into the hollow channel of the CFS (Figure 1B). The rationale of the sequential drug release is built on the unique core/shell helical structure and the different concentrations of alginate gels cross-linking with CaCl_2_. The drug release behavior from hydrogel was generally affected by the concentration and cross-linking density of hydrogel [30,31]. Therefore, we first investigated the effect of the concentration and cross-linking density of alginate on drug release. Doxorubicin (DOX), as the model drug, was loaded in 7 wt% alginate, which was then printed into the core part of the core/shell fibers (drug 1). After cross-linking with different concentrations of CaCl_2_, the drug release from the CFS was investigated. As expected, the drug release was reduced with the increase of the cross-linking density (the increase of the concentration of CaCl_2_). When the CFS was cross-linked by 1 M CaCl_2_, drug 1 (DOX) achieved a sustained release over 175 h (Figure 1C). 

To investigate the effects of the concentration of alginate on drug release from the shell part of the core/shell fiber (drug 2), 9%, 12%, and 15% alginate inks (wt%) carrying the same amount of DOX were used to print the CFS, respectively. As shown in Figure 1D, the DOX release rate from the 15% alginate group was slightly slower than that of the other two groups. Additionally, the concentration of alginate showed a significant effect on drug release from the channel of the CFS (drug 3). As presented in Figure 1E, the drug release was much slower from 9 wt% alginate than that from 5 wt% and 7 wt% alginate. Surely, the highly concentrated alginate inks contributed to forming a much-dense gel network, which reduced the diffusion rate of the drugs from the gel [32]. Meanwhile, the dense wall of the shell fiber could prevent drug leakage from the fiber wall.

Thereafter, the triple drug-loaded CFS could be created by loading drugs in different areas of the CFS to achieve the sequential release of drugs. According to the results (Figure 1F), DOX loaded in the shell part of the CFS (drug 2) achieved the fastest release, while DOX loaded in the channel of the CFS (drug 3) showed the slowest release. The main reason is that the DOX at the shell layer of the fibers was released freely without the preservation effect of the dense shell layer. However, due to the physical blockage by the shell layer, the loaded DOX in the core of fibers and the channel of CFS could only be released from the open ends. The release rate and sustained duration could be controlled by the concentration of alginate, the size, and the number of open orifices in the fibers and CFS. 

Furthermore, it is interesting to note that not only hydrophilic drugs (such as DOX) but also hydrophobic drugs could be loaded in the CFS to achieve long-term release. For instance, Dexamethasone (DEX), a hydrophobic drug, was selected to load into PLGA microspheres, which were then mixed with alginate inks to print into the CFS. From SEM images (Figure 1G), it can be observed that the DEX-loaded microspheres with particle sizes of 3–10 μm have ideal monodispersity and no aggregation among the microspheres. The DEX-loaded PLGA microspheres were embedded into the alginate shell part of the CFS homogeneously (Figure 1H). In this system, drugs showed a long-term sustained release over 100 days (Figure 1I), which was attributed to the slow degradation of the PLGA microspheres and the protection of the concentrated alginate inks.

### 3.2. NIR-Triggered Drug Release System

**Figure 2:**
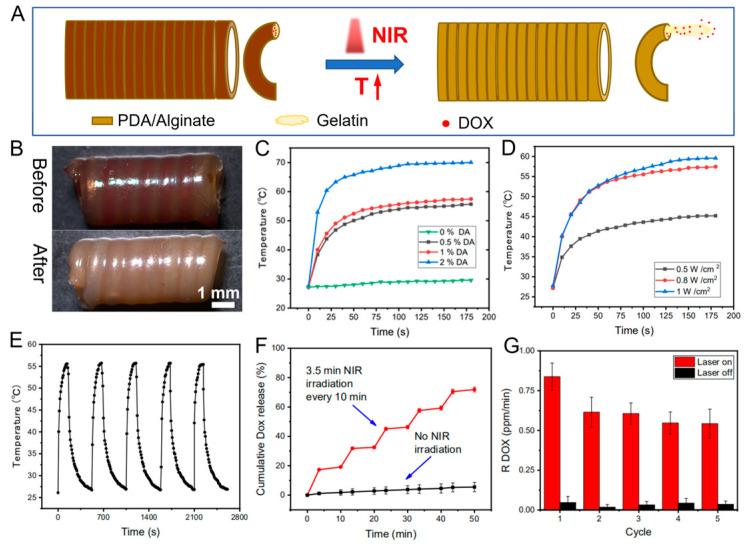
The schematic indicating the NIR-triggered on-demand drug release from prepared CFS (**A**), and the photographs of the DOX-loaded CFS before and after drug release (**B**); heating curves of the CFS with different concentrations of PDA (0.8 W cm^−2^, 808 nm NIR) (**C**) under different laser power (1% PDA) (**D**); photo-thermal conversion cycling test of the CFS (**E**); DOX release from the CFS with and without laser irradiation (0.8 W cm^−2^) (**F**); and DOX release rate with and without laser irradiation *n* = 4 (**G**).

In this system, the CFS is not only suitable for sequential delivery of triple drugs but also can serve as a platform for stimuli-responsive drug release. Herein, polydopamine (PDA) with good photo-thermal conversion efficiency was introduced into the shell layer of the core/shell fibers, endowing the shell part with photo-thermal effects. Gelatin, with a feature of thermosensitive sol-gel transition, as the drug loading matrix was printed into the core part of the core/shell fibers. Once the fibers were irradiated by the NIR laser, PDA could cause a temperature rise in the system, which simulated the diffusion of drug-loaded gelatin from the core part of the fibers, achieving drug release (Figure 2A,B). After the laser was withdrawn, gelatin underwent a sol-gel transition, and drug-loaded gelatin could be retained in the fibers. Firstly, the photo-thermal effects of the core/shell fibers prepared with different amounts of PDA were investigated under NIR irradiation with different power densities. The data demonstrates that the temperature rapidly reached 50 °C, 53 °C, and 65 °C corresponding to the PDA contents of 0.5%, 1%, and 2% (*w*/*v*) (0.8 W/cm^2^, 1 min), respectively (Figure 2C). The temperature of the system was increased with the increase of the PDA amount in the CFS, as well as the irradiation power density. Additionally, the temperature of the system was increased with the increase of the laser power. For instance, the temperature of the system rapidly reached 42 °C and 53 °C under the irradiation of 0.5 W/cm^2^ and 0.8 W/cm^2^ laser for 1 min, respectively (Figure 2D). Furthermore, after five cycles of laser irradiation, the core/shell fibers manifested excellent photo-thermal stability (Figure 2E), indicating the capability of the system for repetitive on-demand drug release. Collectively, all results demonstrated that the PDA mixed CFS displayed excellent photo-thermal conversion efficiency and the potential for NIR-triggered drug delivery. 

Then, the in vitro drug release was evaluated by irradiating CFS (0.8 W/cm^2^ 808 nm NIR) once (3.5 min) every 10 min five times. According to the result (Figure 2F), the drug was significantly released under laser irradiation, while few DOX were released in the absence of laser irradiation. Because of the preservation effect of the dense shell layer and the stable gel state of gelatin in the core part, drugs entrapped in the fibers with less diffusion. Once the core/shell fiber was irradiated with laser, the gel-sol transition of the gelatin resulted in the outflow of the gelatin from the fibers, which then led to the subsequent drug release from the loosened hydrogel networks [33]. The unique “stepped” profile of the drug release under NIR irradiation showed that drug release from the system could be triggered by NIR irradiation, and the amount of released drug was highly dependent on the duration of NIR exposure. The results (Figure 2G) indicated that the released amount of drugs within the five consecutive irradiation cycles was kept at a constant level, suggesting that NIR photo-thermal triggered precise drug release can be achieved by alternatively switching on/off the NIR laser.

### 3.3. Cell Experiment

**Figure 3:**
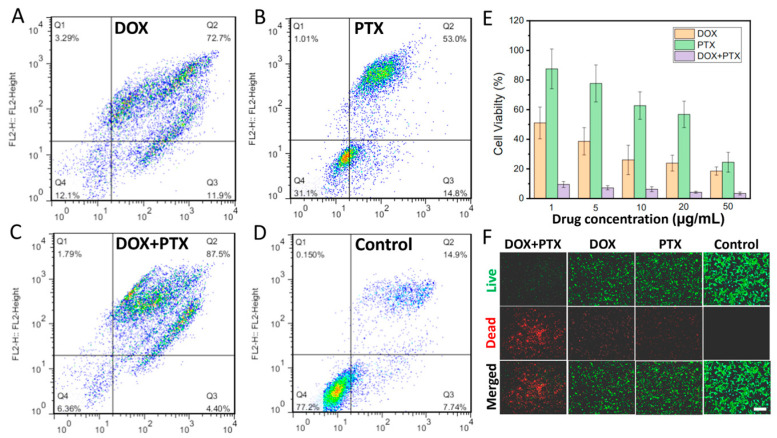
The flow cytometry of groups with CFS loading DOX (**A**), PTX (**B**), DOX + PTX (**C**), and without treatment (**D**). The cell viability of the groups with different treatments *n* = 4 (**E**). Live/dead staining of tumor cells (**F**). (Scale bar: 100 μm).

The potential application of the dual or triple drug-loaded CFS for cancer treatment was also investigated by loading DOX and PTX into the CFS as drug 1 and drug 2, respectively. Firstly, the in vitro cell experiment was performed. The results of flow cytometry illustrated that compared to the other groups, DOX and PTX dual drugs loaded CFS could kill more cells (Q2, 87.5%) (Figure 3A–D), suggesting a huge potential of the multi-drug delivery system for combination therapy. Similarly, as illustrated in Figure 3E, the cell viability in the group of the dual drugs loaded CFS was dramatically decreased at all given concentrations, and over 80% of the cells were dead. However, only at high concentrations could the sole drug-loaded CFS (only DOX or PTX) kill cells effectively. Therefore, a dual drug-loaded system for cancer treatment could significantly reduce the dosing of drugs, minimizing the drug-caused side effects. 

### 3.4. In Vivo Tumor Eradication 

**Figure 4:**
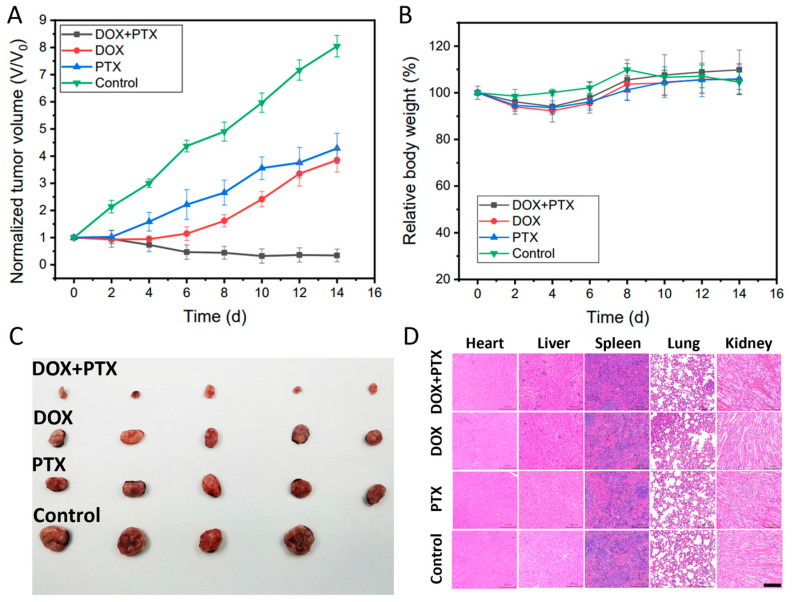
The tumor volume changes curves *n* = 4 (**A**). The body weight curves of mice *n* = 4 (**B**). The digital photograph of tumors collected from the killed mice (**C**). H&E-stained images of major organs (heart, liver, spleen, lung, and kidney) of mice (**D**) (Scale bar: 100 μm).

The in vivo cancer treatment of the dual drug-loaded CFS was examined by implanting the CFS in tumor areas of mice. The tumor-bearing mice were treated as following groups: (1) without any treatment (control); (2) the DOX-loaded CFS, (3) the PTX-loaded CFS, and (4) the DOX + PTX dual drugs loaded CFS. The experiment results showed that the DOX + PTX-loaded CFS represented the best tumor suppression as the tumor growth was completely inhibited compared to the other groups. The groups of only DOX or PTX-loaded CFS exhibited limited tumor inhibition effect, while the tumors in the control group without any treatment grew fast during the following weeks (Figure 4A,C). In addition, there were insignificant changes in the weights of all the mice throughout the experiment (Figure 4B), suggesting that the drugs loaded with CFS had negligible side effects on the mice. Hematoxylin and eosin (H&E) staining images of the major organs (heart, liver, spleen, lung, and kidney) did not have observed abnormality (Figure 4D), further confirming the negligible side effect of the CFS drug delivery system. This multi-drug delivery system might provide a platform for localized therapy of tumors by implantation or minimally invasive injection. 

### 3.5. The pH-Responsive Drug Delivery System

**Figure 5:**
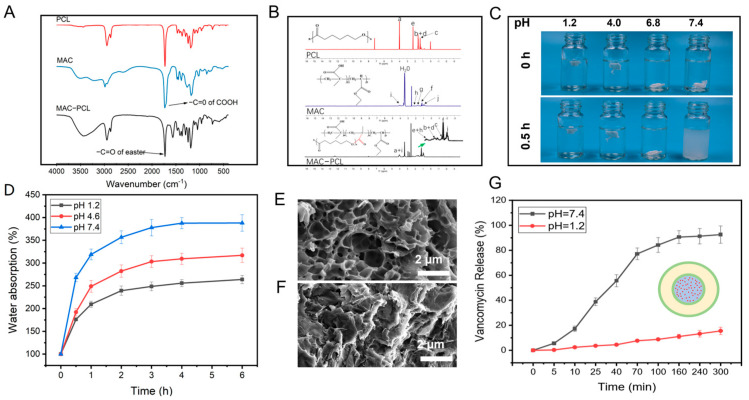
FTIR spectra (**A**) and 1H NMR spectra (**B**) of PCL, MAC, and PCL–MAC; digital photograph of PCL–MAC hydrogel in different pHs for 0 h and 0.5 h (**C**); the swelling ratios curves of PCL–MAC hydrogel in different pHs, *n* = 4 (**D**); the SEM view of the PCL–MAC hydrogel in pH 7.4 (**E**) and 1.2 (**F**) after freeze-dry; the vancomycin release in PCL–MAC hydrogel in pH 7.4 and 1.2 conditions *n* = 4 (**G**).

On-demand drug release responding to exogenous stimuli (such as NIR laser irradiation) was realized using the prepared CFS. Furthermore, endogenous stimuli-responsive release (such as pH value) could also be designed based on this system. For example, a drug-loaded pH-sensitive polymer could be printed in the hollow channel of the CFS to treat specific diseases by triggering drug release responding to pH value change. Herein, we synthesized a pH-sensitive polymer by grafting polycaprolactone onto the methacrylic acid copolymer (PCL-MAC) as the vehicle for drug delivery. FTIR and 1HNMR were applied to characterize the synthetic polymer. In the PCL–MAC FTIR spectrum, there was a wide absorption peak at 3100−3500 cm^−1^ related to the -OH groups of the MAC, and the stretching vibration of the ester bond (–C=O) at 1712 cm^−1^ was stronger, indicating the new ester bond formation (Figure 5A). These data revealed that MAC was grafted to the PCL successfully. As can be seen from Figure 5B, all the characteristic peaks of PCL and MAC could be identified in the 1H NMR spectrum of MAC−PCL, which further confirmed this result. MAC is a pH-sensitive material, so the sol-gel transition of the PCL–MAC is significantly influenced by pH value. After incubation in solution with pH 1.2, 4.0. or 6.8 for 0.5 h, PCL–MAC swelled into the hydrogel, while PCL–MAC gradually dissolved at pH 7.4, revealing a sol state (Figure 5C). The water absorption of PCL–MAC in different pH solutions (pH 1.2, pH 4.0, and pH 7.4) was investigated (Figure 5D). The water absorption of PCL–MAC was increased as the pH value increased from 1.2 to 7.4 and reached equilibrium after 2 h. The pKa of the carboxylic acid of PCL–MAC was about 4.5, so carboxyl acid groups of PCL–MAC tended to dissociate and convert to -COO− at pH > 4.5, breaking the hydrogen bonds between -OH groups of PCL and -COOH groups of MAC, resulting in enhanced solubility of the PCL-MAC. In contrast, when pH < 4.5, the hydrogen bonds increased due to the protonation of the carboxyl groups [20]. Consequently, the compact gel network structure was formed, and the movement and relaxation of the chains within the hydrogel were severely restricted. The SEM analysis on PCL–MAC hydrogels after immersing in pH 7.4 solution (PCL–MAC @ pH 7.4) (Figure 5E) and pH 1.2 solution (PCL–MAC @ pH 1.2) for 0.5h (Figure 5F) were measured, respectively. Compared to the PCL–MAC @ pH 1.2, the PCL–MAC @ pH 7.4 had porous microstructures with sizes around 1–2 µm, which indicated higher water absorption ability. Under the pH 7.4 circumstance, the ionized carboxyl groups (-COO−) were propitious to the opening of the condensation network structure for PCL–MAC. On the contrary, under pH 1.2 conditions, the carboxyl groups were hydrophobic, leading to a dense network structure. It is well known that the pH in the stomach is 1.0–3.0, and in the intestine, it is 5.0–8.0 [34,35]. Therefore, PCL–MAC had the potential for gastrointestinal tract-specific drug delivery. Vancomycin-loaded PCL–MAC could be printed into the channels of the CFS to investigate the pH-dependent drug release. The release of vancomycin from the CFS at different pHs (pH 1.2 and pH 7.4) were evaluated. The data demonstrated that a small amount of vancomycin was released at pH 1.2 after 300 min incubation, while 98% of vancomycin was released after incubating at pH 7.4 for 100 min (Figure 5G). The compact structure prevented the diffusion of the drug at pH 1.2, and the opening of the sol network structure facilitated drug release at pH 7.4. Therefore, it is reasonable to expect that drugs within the system could be protected from degradation caused by gastric acid and subsequently released in the intestinal tract. Thus, this 3D printed system might also be applicable for the treatment of gastrointestinal diseases through oral delivery.

## 4. Conclusions

In this study, we prepared a multi-drug-loaded core/shell fiber system by co-axial 3D printing to achieve sequential release of dual or triple drugs. As a result, drugs loaded in the shell part of the CFS (drug 2) achieved the fastest release, while drugs loaded in the channel of the CFS (drug 3) exhibited the slowest release. The main factors affecting the release rate and duration include the unique core/shell helical structure, the different concentrations of alginate gels, and the size and number of the open orifices in the fibers of CFS. Furthermore, exogenous and endogenous stimuli-responsibility (e.g., laser and pH) could be introduced in this system to achieve stimuli triggered (NIR laser irradiation or pH value change) on-demand drug release. Lastly, the synergistic effect of dual drugs released from the system could effectively kill cancer cells and inhibit the growth of tumors with low dosages. The 3D-printed multi-drug-loaded system is expected to realize the full potential of combination therapies and reveal great promising applications in tissue engineering and localized therapy of cancer or other diseases.

## Data Availability

The data that support the findings of this study are available from the corresponding author, Yuxiao Li, upon reasonable request.
